# Determinants of Prenatal Exposure to Polychlorinated Biphenyls (PCBs) and Polybrominated Diphenyl Ethers (PBDEs) in an Urban Population

**DOI:** 10.1289/ehp.10333

**Published:** 2007-09-27

**Authors:** Julie B. Herbstman, Andreas Sjödin, Benjamin J. Apelberg, Frank R. Witter, Donald G. Patterson, Rolf U. Halden, Richard S. Jones, Annie Park, Yalin Zhang, Jochen Heidler, Larry L. Needham, Lynn R. Goldman

**Affiliations:** 1 Columbia Children’s Center for Environmental Health, Columbia Mailman School of Public Health, New York, New York, USA; 2 Division of Laboratory Sciences, National Center for Environmental Health, Centers for Disease Control and Prevention, Atlanta, Georgia, USA; 3 Department of Epidemiology, Johns Hopkins Bloomberg School of Public Health, Baltimore, Maryland, USA; 4 Department of Gynecology and Obstetrics, Johns Hopkins School of Medicine, Baltimore, Maryland, USA; 5 Department of Environmental Health Sciences, Johns Hopkins Bloomberg School of Public Health, Baltimore, Maryland, USA

**Keywords:** dichlorodiphenyldichloroethylene, dichlorodiphenyltrichloroethane, environmental exposure, epidemiology, fetal exposure, polychlorinated biphenyls, polybrominated diphenyl ethers, prenatal, urban

## Abstract

**Background:**

Recent studies have reported blood levels of polybrominated diphenyl ethers (PBDEs) in the U.S. population. Information about neonatal levels and about the relationship to polychlorinated biphenyls (PCBs) exposures is limited.

**Objectives:**

The objective was to characterize levels and determinants of fetal exposure to PBDEs and PCBs among newborns from Baltimore, Maryland.

**Methods:**

We analyzed umbilical cord blood for eight PBDEs and 35 PCBs from infants delivered at the Johns Hopkins Hospital. Maternal and infant characteristics were abstracted from medical records.

**Results:**

Ninety-four percent of cord serum samples had quantifiable levels of at least one PBDE congener, and > 99% had at least one detectable PCB congener. PBDE concentrations in cord blood were similar to those reported in other studies from North America. Strong correlations were observed within but not across PCB and PBDE classes. Multivariate models showed that many factors independently predicted exposure to BDE-47, BDE-100, and BDE-153 and CB-118, CB-138/158, CB-153, and CB-180. Generally, infants of Asian mothers had lower PBDE and PCB levels, and infants of smokers had higher levels. Increased maternal body mass index was associated with lower levels of PCBs but not PBDEs. Levels of PCBs but not PBDEs were lower in births from married and multiparous mothers. Increased maternal age was associated with higher PCB levels but lower PBDE levels.

**Conclusions:**

Although many of the factors we investigated were independent predictors of both PBDE and PCB levels, in some cases the direction of associations was different. More research is needed to better understand the sources and pathways of PBDE exposure.

Use of polybrominated diphenyl ethers (PBDEs) as flame retardants (FRs) in plastics, including electronic enclosures and poly-urethane used in upholstery cushioning and carpet pads, has increased steadily in the past 30 years (Alaee et al. 2003; de Wit 2002; Hardy 2002b). PBDEs used as additive FRs are more likely to leach over time than reactive FRs, which are chemically bonded to the plastic polymer (Alaee and Wenning 2002; Eriksson et al. 2001; Sjodin et al. 2001a). PBDEs are similar to polychlorinated biphenyls (PCBs) in structure, lipophilicity, persistence, and ability to bioaccumulate (Hardy 2002a; McDonald 2002). Since the 1970s, environmental concentrations of PCBs and other persistent organic pollutants (POPs) have fallen whereas levels of PBDEs have increased exponentially (Alaee and Wenning 2002; Eriksson et al. 2001; Meironyte et al. 1999; Sjodin et al. 2001b). Currently, human PBDE levels are of a magnitude similar to PCB concentrations in some regions (Hites 2004; Petreas et al. 2004). Human levels of PBDEs are higher in North America than in Europe and within a given population, a small fraction (5–10%) of individuals have concentrations far exceeding average levels (Betts 2003; Hites 2004; Schecter et al. 2005; Sjodin et al. 2003; Wilford et al. 2005). Although there have been no studies investigating the health effects associated with PBDEs in humans, there are reports of human exposure levels that may be on par with levels eliciting harmful effects in laboratory animals (McDonald 2005). Animal studies have shown evidence of the disruption of normal endocrine function and neurodevelopmental, hepatic, and reproductive toxicity (Betts 2004, 2005; Johnson-Restrepo et al. 2005).

Currently, there is an incomplete understanding of how humans are exposed to PBDEs. It is reasonable to posit that among those with high concentrations, occupational exposures may be involved in some cases (Sjodin et al. 1999; Thuresson et al. 2005). In the general population, several potential routes of PBDE exposure have been identified: *a*) direct contact with treated products; *b*) dietary, as these lipophilic compounds are stored in the fat of meats, fish, and dairy products (Harrad et al. 2004; Schecter et al. 2004, 2006; Wilford et al. 2005); and *c*) ingestion and to a lesser degree inhalation of PBDE-containing house dust (Jones-Otazo et al. 2005; Stapleton et al. 2005; Wilford et al. 2005; Wu et al. 2007) Only a few studies have been able to explore individual determinants of PBDE body burdens in much detail (Morland et al. 2005; Wu et al. 2007).

Levels of PBDEs (as well as PCBs and organochlorine pesticides) measured in adipose tissue, blood, or milk can be used to assess exposures (Sjodin et al. 2003). Infants and fetuses are sensitive subpopulations to consider, because these stages of development may be especially susceptible to effects of chemical exposures (Howdeshell 2002). Toxicologic models have indicated that immature animals have slower metabolism and excretion of PBDEs; approximately 50% of the administered 2,2′,4,4′-tetraBDE (BDE-47) dose is excreted in the first 24 hr in adult animals compared with 31% and 41% in animals dosed at 10 and 22 days after birth (Staskal et al. 2006). Measuring PBDEs in cord blood serum is noninvasive, using a blood sample that is otherwise usually discarded, and is a direct measure of prenatal exposure. The estimated half-lives of these compounds in humans are long enough—BDE-47 1.8–3.0 years, 2,2′,4,4′,5-pentaBDE (BDE-99) 2.9–5.4 years, and 2,2′,4,4′,5,5′-hexaBDE (BDE-153) 6.5–11.7 years (Geyer et al. 2004)—that cord blood concentrations reflect exposure through the course of pregnancy. Such levels also provide a reasonable estimate of maternal serum concentrations (Mazdai et al. 2003).

The principal objective of this investigation was to characterize fetal exposure to PBDEs and selected PCBs among newborns from Baltimore, Maryland, via the assessment of concentrations in umbilical cord serum. We explored demographic characteristics and attributes to identify determinants and to explain variations in exposure levels and patterns within this largely urban and African-American population.

## Materials and Methods

### Study design and population

We conducted a cross-sectional study of newborn deliveries at the Johns Hopkins Hospital in Baltimore, Maryland. In this study we examine the associations between exposures to a number of toxic chemicals, thyroid hormone levels, and birth outcomes; determinants of PCB and PBDE exposure are presented here. We collected umbilical cord blood from a sample of women presenting at the Johns Hopkins Hospital for delivery of singleton births from 26 November 2004 to 16 March 2005. Samples were excluded if the family wished to archive cord blood or if samples yielded < 5.2 mL of serum. During the study period, of 597 singleton births delivered, 341 cord blood samples were collected, of which 300 had sufficient serum volume for laboratory analyses. A brief survey of hospital personnel revealed that specimens were missed mainly because of complications during delivery, premature birth and/or small size of the infant resulting in small cord blood volume, and logistical factors such as understaffing. This study had the approval of the Maternal and Fetal Research Committee in the Department of Gynecology and Obstetrics and the Institutional Review Board (IRB) at the Johns Hopkins University (JHU) School of Medicine, and a Health Insurance Portability and Accountability (HIPAA) waiver. The requirement for informed consent was waived by the IRB because drawing blood from the placenta involves no more than minimal risk to the subjects, and the biosamples collected would otherwise have been discarded. Strict safeguards preserving patient confidentiality were established. Members of a community advisory committee, who were selected for their specific knowledge and expertise on important child health concerns in Maryland, had the opportunity to learn about and comment on this study.

### Data collection

Nurses or physicians who routinely collect cord blood specimens for clinical purposes obtained umbilical vein blood using a syringe immediately after newborn delivery but before the delivery of the placenta (Witter et al. 2001). The cord blood was transferred from the syringes to silicon-coated (PBDE-free) vacutainers that were stored for a maximum of 3 hr at 4°C. The vacutainers were centrifuged at 2,400 × *g* for 15 min, and serum was pipetted into pre-screened amber glass vials with Teflon-lined screw caps, which were immediately stored at −80°C (Sjodin et al. 2004b). Frozen specimens were transferred on dry ice to the Centers for Disease Control and Prevention (CDC) for laboratory analyses.

Details regarding the analysis of the serum samples for PBDEs and PCBs are described elsewhere (Sjodin et al. 2004b). Briefly, the samples were fortified with ^13^C-labeled internal standards and formic acid and water for denaturation and dilution using a Gilson 215 liquid automated handler (Gilson Inc., Middleton, WI). The samples were extracted using the Rapid Trace (Caliper Life Sciences, Hopkinton, MA) modular solid phase extraction (SPE) system. Lipids were co-extracted using the SPE system and were removed on a silica:silica/sulfuric acid column. The final analytic determinations were performed using a gas chromatography isotope dilution high resolution mass spectrometry employing a MAT95XP (ThermoFinnigan MAT, Bremen, Germany) instrument. The serum lipid concentration was determined by use of commercially available test kits from Roche Diagnostics Corp. (Indianapolis, IN) for the quantitative determination of total triglycerides (product no. 011002803-0600) and total cholesterol (product no. 011573303-0600). Final determinations were made on a Hitachi 912 chemistry analyzer (Hitachi, Tokyo, Japan).

A total of 297 of the 300 cord serum samples were successfully analyzed. The limits of detection (LOD) were defined both in relation to the method blanks and the instrumental detection limit (Sjodin et al. 2004b). We analyzed the serum samples for the following PBDE congeners (International Union of Pure and Applied Chemistry numbers): 2,4,4′-tribromodiphenyl ether (BDE-28), 2,2′,4,4′-tetraBDE (BDE-47), 2,2′,3,4,4′-pentaBDE (BDE-85), 2,2′,4,4′,5-pentaBDE (BDE-99), 2,2′,4,4′,6-pentaBDE (BDE-100), 2,2′,4,4′,5,5′-hexaBDE (BDE-153), 2,2′,4,4′,5,6′-hexaBDE (BDE-154), and 2,2′,3,4,4′,5′,6-heptaBDE (BDE-183). We analyzed 21 samples for decaBDE (BDE-209); all of these specimens had a concentration below the LOD. Because of this observation, the concentration of BDE-209 was not determined in remaining specimens. The median LOD for BDE-209 was 3.0 ng/g lipid, and the range was 2.0–3.7. The samples were also analyzed for 35 PCB congeners [the full list is available in Supplemental Material, Table 1 (online at http://www.ehponline.org/members/2007/10333/suppl.pdf)]; the results of the following four congeners are presented here: 2,3′,4,4′,5-pentaCB (CB-118), 2,2′,3,4,4′,5′-hexaCB/2,3,3′,4,4′,6-hexaCB (CB-138**/**158, co-elution), 2,2′,4,4′,5,5′-hexaCB (CB-153), and 2,2′,3,4,4′,5,5′-heptaCB (CB-180). The specimens were also analyzed for 9 persistent pesticides, of which only 2,2-bis(4-chlorophenyl)-1,1-dichloro-ethene (*p,p*′-DDE), the primary degradation product of *p,p*′-DDT (dichlorodiphenyl-trichloroethane), is reported here. We measured cotinine concentrations in cord blood by use of liquid chromatography in conjunction with atmospheric pressure ionization tandem mass spectrometry (Bernert et al. 1997).

Demographic information and biological measures were abstracted from the maternal medical records by two study personnel, and a 10% random sample was re-reviewed by study clinicians. Additional information was obtained from forms completed by the nursing staff at the time of delivery. Age, education, marital status, and parity were self-reported. Self-reported maternal race was also recorded because it appeared in the medical records as one of three categories: African American, Caucasian, and Asian. No further information was available regarding length of residency in the United States or a further ethnic breakdown within these three race categories. Smoking status was defined using a combination of the maternal medical record and cord serum cotinine concentrations; levels > 10 ng/mL were categorized as maternal active smoking (CDC 2005). If the clinical record indicated that the mother reported smoking at any time during pregnancy, she was considered an active smoker, regardless of the cotinine concentration. Maternal street addresses were geocoded by GeoLytics Inc. (East Brunswick, NJ), who also provided census information at the block-group level. Once data collection was completed, medical record numbers and addresses were deleted from study databases and files. Geographic locations were maintained at the block-group level.

### Data analyses

PCB and PBDE distributions were log-normally distributed; medians and interquartile ranges (IQRs) are presented, and concentrations were log-transformed for regression analyses. To confirm that the distributions conformed to the assumptions of normality, (P-P) plots (graphical representations of the transformed PCB/PBDE distributions compared with a normal distribution) were used. For analytes below the LOD, the value imputed is the LOD divided by the square root of 2. PCB and PBDE concentrations were expressed as nanograms per gram lipid.

We used Spearman correlation coefficients to explore bivariate relationships between various PBDEs and organohalogen compounds. PCBs 118, 138/158 (co-eluting congeners), 153, and 180 were selected for further analysis on the basis of their proportional contribution to the ∑PCB concentration and having a large proportion of the samples with detectable levels. Only PBDEs with ≥ 60% detectable (BDE-47, BDE-100, and BDE-153) were evaluated further. We developed univariate and multivariate linear regression models for these congeners. To avoid excluding observations in multivariate analyses, we substituted the median for missing values [4 median substitutions for maternal education, 7 for timing of first prenatal visit, 9 for weight gain during pregnancy, 4 for median household income, and 11 for body mass index (BMI)]. Also, “high” exposure groups were defined as individuals in the highest 10% of the exposure distribution for BDE-47, BDE-153, CB-153, and CB-180 concentrations, respectively; this end point was modeled using multivariate logistic regression models. We evaluated statistical differences by exposure group by Student’s *t*-test and Fisher’s exact test. All statistical analyses were conducted using STATA, version 8.0 (StataCorp., College Station, TX).

## Results

Of the 300 samples sent for PCB and PBDE analyses, 297 successfully underwent sample preparation and quantification. The 41 infants with insufficient serum quantity were more likely to be the first born (61% vs. 42%), preterm (24% vs. 13%), and/or low birth weight (22% vs. 11%). Concentrations (nanograms per gram lipid) of analyzed PBDE and selected PCB congeners are given in Table 1, with the percentage of results above the LOD. Ninety-four percent of the cord samples contained at least one detectable BDE congener, and > 99% had at least one detectable PCB congener. Among the eight BDE congeners monitored, the tetrabrominated BDE-47 was the dominant congener by weight, accounting, on average, for 51.2% of the ∑PBDE concentration. In order of decreasing abundance, BDE-47 was followed by BDE-99 (12.0%), BDE-153 (8.2%), and BDE-100 (8.1%), with BDE-28, BDE-85, BDE-154, and BDE-183 each accounting for < 4% of the ∑PBDE concentration. We calculated Spearman correlations between pairs of selected PCB and PBDE congeners and DDE. In general, the correlations were the highest within each compound class, whereas PCB and PBDE congeners were not highly correlated with each other. PCBs but not PBDEs were correlated with serum DDE levels [see Supplemental Material, Table 2 (online at http://www.ehponline.org/members/2007/10333/suppl.pdf)].

In this population, the mean (± SD) maternal age was 25.9 ± 6.6 years. Most (70%) were African American, with 21% Caucasian and 8% Asian. Most (66%) of the mothers were unmarried, and a large proportion (29%) had less than a high school education. Use of standardized categories of BMI showed that almost half of the mothers were overweight or obese before pregnancy (National Institutes of Health 1998). On the basis of self-reported smoking status, 15% of women smoked tobacco during the pregnancy. However, after validating self-reported tobacco use with cord serum cotinine levels, 19% were classified as active smokers during pregnancy. Nearly 70% resided within the Baltimore city limits.

**Figure 1:**
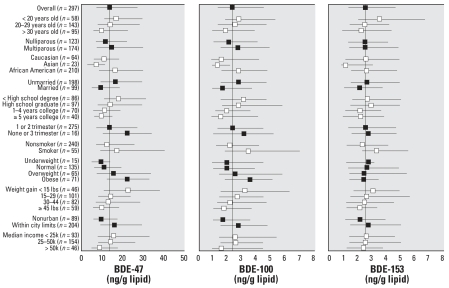
Univariate (unadjusted) relationships between lipid-adjusted PBDE levels in cord blood serum and sample characteristics. The median and QR are represented by error bars; the vertical line is the overall median for this population. Black and white symbols distinguish between variables.

**Figure 2:**
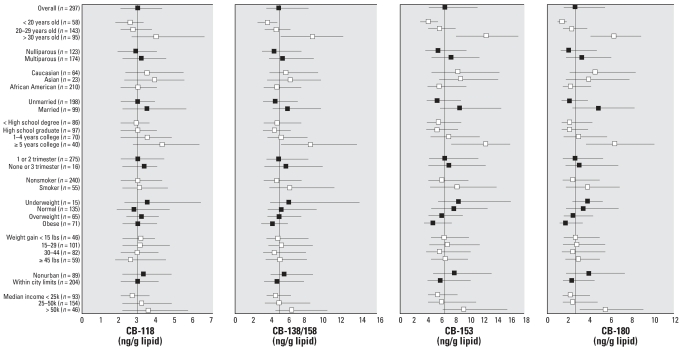
Univariate (unadjusted) relationships between lipid-adjusted PCB levels in cord blood serum and sample characteristics. The median and IQR are represented by error bars; the vertical line is the overall median for this population. Black and white symbols distinguish between variables.

Median and interquartile ranges of lipid-adjusted PBDE and PCB concentrations are shown in Figures 1 and 2, stratified by demographic variables. These univariate analyses show general exposure patterns for the two sets of compounds. The relationships between the covariates presented in Figures 1 and 2 and cord serum concentrations were evaluated by use of multivariate linear regression models (Tables 2 and 3). The adjusted regression coefficients were compared with unadjusted regression coefficients to evaluate confounding.

In a multivariate model of cord BDE-47, younger maternal age, less pregnancy weight gain, having ≥ 5 years of college education, and being classified as obese prepregnancy were independently associated with increased cord blood levels (Table 2). For BDE-100, only maternal smoking during pregnancy was predictive of higher cord blood levels in a multivariate model. For BDE-153, the multivariate model indicated that younger maternal age, less weight gain during pregnancy, and smoking during pregnancy were associated with higher cord blood levels. Asian infants had lower mean cord levels of BDE-47 and BDE-153. The multivariate models indicate that the univariate relationships between some of the independent variables and PBDE exposure were confounded. For example, for BDE-47, a number of variables—maternal race, median household income in the neighborhood, marital status, and residence in or outside of the Baltimore city limits—were no longer associated with cord serum levels after controlling for maternal age, weight gain during pregnancy, education, and BMI.

In the case of PCB serum levels (Table 3), both univariate and multivariate linear regression models for CB-118, CB-138/158, CB-153, and CB-180 indicated that maternal age was positively associated with umbilical cord PCB levels. For CB-118, in addition to maternal age, the multivariate model indicated that infants of unmarried mothers and mothers having ≥ 5 years of college education had increased exposure. In the case of CB-138/158, infants of older mothers, unmarried mothers, and mothers who smoked during pregnancy had higher mean exposure levels. Infants of Asian and obese mothers had lower mean cord levels of CB-138/158. For both CB-153 and CB-180, the multivariate models indicated that besides increasing maternal age, lower birth order (parity), being unmarried, and maternal smoking during pregnancy were independently associated with higher cord levels. Infants of Asian and obese mothers had lower mean cord serum level of CB-153 and CB-180. Similar to PBDEs, the PCB multivariate models suggest that some of the univariate relationships were confounded.

Within our population, subgroups of high exposure were identified as described above. Ten individuals were in the highest decile of both BDE-47 and BDE-153, and 23 individuals were in the highest of both CB-153 and CB-180. Only one individual was in the highest decile of all four congeners considered in this analysis. The odds of being in the highest decile of exposure were examined for these four congeners using multivariate logistic regression and the same independent variables as presented in Tables 2 and 3. Although the 95% confidence intervals (CIs) are wide, infants of mothers who smoked during pregnancy [odds ratio (OR) = 4.10; 95% CI, 1.51–11.10] and those whose mothers had completed ≥ 5 years of post–high school education (OR = 11.51; 95% CI, 1.36–97.66) were significantly more likely to be in the high BDE-47 exposure group. There were no covariates significantly associated with being in the high BDE-153 exposure group. Infants of older mothers (OR = 1.42, 95% CI, 1.26–1.61; OR = 1.44, 95% CI, 1.26–1.64) and mothers with a prior childbirth (OR = 6.06, 95% CI, 1.57–23.45; OR = 6.76, 95% CI, 1.68–27.16) were more likely to be in the high CB-153 and CB-180 exposure groupings, respectively.

## Discussion

We measured PBDE and PCB concentrations in cord serum of 297 neonates born at an inner-city referral hospital. To date there are few data with regard to exposures in inner-city, largely African-American populations. Although concentrations of individual PBDEs and PCB congeners were correlated within classes, they were not well correlated across classes. Some studies have reported positive correlations between these two classes of compounds in humans (Kalantzi et al. 2004) and in fish (Hale et al. 2001; Hayward et al. 2004). Others, including ours, report no such association (Bradman et al. 2007; Johnson-Restrepo et al. 2005; Thomas et al. 2005). Although PBDEs are found in a variety of household and consumer products, PCBs no longer are manufactured for use, and most existing uses have been phased out. This is consistent with the finding that PCBs, but not PBDEs, are correlated with *p,p*′-DDE levels. *p,p*′-DDE is the degradation product of *p,p*′-DDT, which has not been actively used in the United States for decades. Differences in PCB and PBDE toxicokinetics may also play a role (Johnson-Restrepo et al. 2005). Generally, as well as in our study, the distribution of PBDEs in humans is log-normally distributed such that approximately 5% of the sample has PBDE levels that are 3–5 times higher than the median (Jones-Otazo et al. 2005; She et al. 2004; Wilford et al. 2005; Wu et al. 2007). Of these, very few were also highly exposed to PCBs, an observation that may indicate that differing routes of exposure or temporal differences in patterns of use are responsible for the highest levels.

To date, two other published studies have reported PBDE levels in cord blood: one in Sweden and one in Indiana (USA) (Mazdai et al. 2003), both in 2001. Although these studies are much smaller than ours, the Indiana study reported similar concentrations of PBDEs in cord blood (median ∑PBDE, 39 ng/g serum lipid; range, 14–460 ng/g) (Mazdai et al. 2003). Our concentrations were more than an order of magnitude higher than the Swedish levels (median ∑PBDE, 1.7 ng/g serum lipid; range, 0.5–4.3 ng/g) (Guvenius et al. 2003). An additional study that evaluated the concentration of PBDEs in human fetal livers (*n* = 11) reported lower concentrations (median ∑PBDE, 15.2 ng/g lipid; range, 4.0–98.5 ng/g) than those measured in this study (Schecter et al. 2007). Levels of PBDEs in cord blood are roughly equivalent to those found in maternal blood on a lipid-adjusted basis (Mazdai et al. 2003), and PBDEs measured on a per-lipid basis may be compared across matrices. PBDE levels in human milk in two U.S. studies are similar to what was measured in our study population on a lipid weight basis (Schecter et al. 2003; Wu et al. 2007). From these comparisons, albeit limited because of different sets of congeners contributing to the total exposure (∑ measurement) in each study, we conclude that the levels found in this study are consistent with U.S. levels reported elsewhere and are much higher than levels in Sweden (Bradman et al. 2007; Hites 2004; Wu et al. 2007).

In most human samples, BDE-47 has been identified as the dominant congener in terms of concentration (Hites 2004; Schecter et al. 2007; Sjodin et al. 2001b). However, there are some populations (mainly in Europe) and a small proportion of individuals within U.S. populations in which BDE-153 is instead the dominant PBDE congener in humans (Covaci et al. 2002; Naert et al. 2006; She et al. 2004; Thomas et al. 2005; Wu et al. 2007). This second pattern is present among 5% of our population. This difference may reflect variable exposure pathways or toxicokinetics or, alternatively, differences in diet among individuals from countries or ethnic groups where BDE-47 is not the dominant congener in foods (Covaci et al. 2002).

Several factors independently predicted exposures to PCBs and PBDEs in multivariate models. The direction of some of these associations was surprisingly different between these two compound classes. PBDEs tended to decrease with older maternal age, whereas PCBs consistently increased. Women who were obese before pregnancy had higher BDE-47 levels. However, for CB-138/158, CB-153, and CB-180, prepregnancy obesity was associated with lower exposure levels. Asians generally had lower PCB and PBDE levels than Caucasians. In addition to differences in exposure routes and pathways, immigration patterns (including the number of years since immigrating to the United States) may partly explain these differences. However, these data were not available for analysis in this study.

The observed positive association between maternal age with PCB levels was expected. In contrast, maternal age was independently negatively associated with each and all PBDE congeners. The levels of PBDEs are still increasing or have been increasing until recently in the general U.S. population, making the influence of accumulation with age less important (Sjodin et al. 2004a). In this way, if most of the exposure to high levels of PBDEs has occurred more recently in time, a person’s length of time with high exposure may be independent of age. Another possible explanation is that PBDEs have shorter half-lives compared with PCBs (Ogura 2004). Other studies have not observed a relationship between age and PBDE concentrations, independent of parity (Schecter et al. 2003; Sjodin et al. 1999, 2003). However, the study by Schecter et al. (2005), which included men and a broader age range, reported a “suggested” inverse association between PBDE levels and age. Among our population, the inverse association with maternal age and PBDEs corresponds to a 12-ng/g lipid decrease in predicted average BDE-47 concentrations over the entire age range in the study (14–43 years). Although the maternal age range in this study covers only one generation, there is the possibility of a behavioral cohort effect, such that younger women may have more contact with consumer products containing PBDEs or have higher consumption of PBDE-containing foods. An association with diet is also suggested by the higher levels among women with obese prepregnancy BMI, although this may reflect lifestyle differences as well.

As expected, multivariate models indicate that body burden of PCBs (CB-153 and CB-180) decreased with increased maternal parity. That is consistent with the notion that pregnancy is one of the major routes of elimination for PCBs as well as other lipophilic POPs (Barr et al. 2005). However, we found no evidence for a decrease in PBDE levels with increased numbers of pregnancy in our study. Lactation is another mechanism for the excretion of PCBs and PBDEs. In this study, information about previous lactation history was unavailable.

Dilution of organohalogen levels via weight gain is another mechanism that could explain lower levels in subjects with a higher BMI. In our population, consistent with other studies, women with very high (obese) prepregnancy BMIs consistently had lower lipid-adjusted PCB levels, and very underweight women tended to have higher PCB levels than did women with normal prepregnancy weight (Glynn et al. 2007). However, such a “fat dilution” phenomenon was not observed for PBDEs; if anything, obese women had higher exposure levels (in the case of BDE-47 and BDE-100). Conversely, reduced weight gain during pregnancy tended to be associated with higher levels of exposure to PBDEs. A study examining the effect of the commercially available DE-71 mixture (consisting of primarily tetra and penta PBDE congeners) in rats did not find an association between weight gain during pregnancy and exposure (Zhou et al. 2002). In this cross-sectional study, it is not possible to determine whether reduced weight gain during pregnancy is a predictor or the consequence of high concentrations of PBDEs.

Smoking during pregnancy was associated with higher cord blood levels of both PCBs and PBDEs. Smokers have increased hand-to-mouth behavior that can increase oral ingestion of contaminated dust in the environment among smokers or altered mucociliary clearance processes in the upper airways of smokers, potentially leading to ingestion of mucus-containing inhaled PBDE particles (Phillips et al. 2003). However, particle ingestion would not explain why smokers also have elevated levels of PCBs, which are not typically found in dust. Smoking may also be associated with other lifestyle factors (including diet) which we were not able to evaluate in this study. Alternatively, smoking alters PCB and PBDE exposure levels via alteration in either maternal or fetal hepatic enzymes (or both). Certainly, smoking induces cytochrome P450 1A2, which has been shown to be involved in metabolism of mono-*ortho* PCBs (Ayotte et al. 2005), such as CB-118, which is the one PCB that was not associated with smoking in our study. This, however, would not explain why levels of the di-*ortho* PCBs such as CB-153 and CB-180 as well as PBDE levels were higher among smokers. Though not readily explained, these associations were consistent and need further study.

In this study, we examined individual and spatial determinants of PBDE exposure that might explain the wide distribution of blood levels evident in this population. Although many of these factors were significant predictors of exposure, overall, most of the variation was unexplained, implying that other unmeasured factors account for this variation. These factors may be related to differential exposure (i.e., dietary differences or differences in micro-environments), which we were unable to collect in this population. It is also possible that the wide variation detected in cord blood serum levels might be related to interindividual genetic differences that impact the ability to absorb, metabolize, or excrete these compounds.

## Figures and Tables

**Table 1:** Lipid-adjusted concentrations and congener distributions of PBDEs and selective PCBs (and DDE for comparison) in umbilical cord blood serum (*n* = 297).

Congener	Percent > LOD	Median LOD (ng/g serum lipid)	Median (ng/g serum lipid)	Range (ng/g serum lipid)	Congener distribution^a^ (median %)
BDE-28	29.6	1.1	0.9	ND–9.8	3.0
BDE-47	90.2	1.3	13.6	ND–311.2	51.2
BDE-85	15.8	1.3	1.1	ND–12.9	3.9
BDE-99	46.5	2.1	4.3	ND–83.4	12.0
BDE-100	64.5	1.2	2.3	ND–77.0	8.1
BDE-153	60.3	1.3	2.6	ND–154.3	8.2
BDE-154	6.1	1.1	0.9	ND–10.1	3.0
BDE-183	7.1	1.1	0.9	ND–13.2	3.3
CB-118^b^	95.6	1.1	3.0	ND–97.5	6.3
CB-138/158	98.0	1.1	4.9	ND–60.2	10.9
CB-153	99.7	1.1	6.4	ND–72.4	13.9
CB-180	89.6	1.1	2.6	ND–68.8	5.8
DDE	100.0	1.1	53.5	3.9–7,710	NA

ND, not detected.

a PBDE congener distribution based on the proportional contribution of 8 congeners; PCB congener distribution based on the proportional contribution of 35 congeners.

b Dioxin-like PCB.

**Table 2:** β coefficients, indicating change in (ln)PBDE concentrations, from univariate and multivariate^a^ regression models for selected PBDEs.

	BDE-47		BDE-100		BDE-153	
	β	Adjusted β (95% CI)	β	Adjusted β (95% CI)	β	Adjusted β (95% CI)
Maternal age (per 5 years)	−0.76**	−0.14* (−0.25 to −0.02)	−0.10**	−0.10 (−0.21 to 0.00)	−0.13**	−0.15** (−0.26 to −0.04)
Maternal weight gain^b^ (per 10 lb)	−1.09**	−0.08* (−0.14 to −0.01)	−0.06*	−0.04 (−0.10 to 0.02)	−0.07*	−0.07* (−0.13 to 0.00)
Median household income^c^ (per $10,000)	−0.10**	−0.03 (−0.10 to 0.04)	−0.07**	−0.01 (−0.08 to 0.06)	−0.02	0.04 (−0.03 to 0.11)
Parity (reference nulliparous, *n* = 123)									
Multiparous (*n* = 174)	0.20	0.19 (−0.06 to 0.44)	0.22*	0.20 (−0.04 to 0.44)	0.06	0.13 (−0.12 to 0.38)
Race (reference Caucasian, *n* = 64)									
Asian (*n* = 23)	−0.56**	−0.70** (−1.17 to −0.23)	−0.34	−0.40 (−0.85 to 0.05)	−0.53*	−0.59* (−1.06 to −0.12)
African American (*n* = 210)	0.40**	0.04 (−0.32 to 0.40)	0.36**	0.08 (−0.27 to 0.42)	0.13	−0.06 (−0.42 to 0.29)
Marital status (reference unmarried, *n* = 198)									
Married (*n* = 99)	−0.51**	0.00 (−0.38 to 0.38)	−0.34**	0.14 (−0.22 to 0.50)	−0.26*	0.10 (−0.27 to 0.48)
Maternal education^d^ (reference < high school, *n* = 86)									
High school graduate (*n* = 101)	−0.16	0.04 (−0.24 to 0.32)	−0.01	0.16 (−0.11 to 0.42)	−0.01	0.18 (−0.09 to 0.46)
1–4 years college (*n* = 70)	−0.49**	0.06 (−0.31 to 0.43)	−0.34*	0.08 (−0.26 to 0.43)	−0.30*	0.09 (−0.27 to 0.46)
≥ 5 years college (*n* = 40)	−0.54**	0.57* (0.04 to 1.11)	−0.41*	0.37 (−0.14 to 0.88)	−0.28	0.33 (−0.21 to 0.86)
Prenatal care^e^ (reference 1st or 2nd trimester, *n* = 281)									
None or 3rd trimester (*n* = 16)	0.36	0.23 (−0.27 to 0.73)	0.11	0.02 (−0.46 to 0.49)	0.07	0.05 (−0.44 to 0.55)
Smoking during pregnancy (reference nonsmoker, *n* = 242)									
Smoker (*n* = 55)	0.30*	0.22 (−0.07 to 0.51)	0.42**	0.40** ( 0.12 to 0.68)	0.42**	0.42** ( 0.13 to 0.71)
BMI^f^ (kg/m^2^) (reference normal, *n* = 135)									
Underweight (*n* = 11)	0.43	0.55* (0.06 to 1.04)	0.05	0.16 (−0.31 to 0.63)	−0.01	0.16 (−0.33 to 0.65)
Overweight (*n* = 65)	0.68*	0.70** (0.18 to 1.22)	0.31	0.34 (−0.16 to 0.84)	0.04	0.11 (−0.41 to 0.63)
Obese (*n* = 71)	1.02**	0.93** (0.40 to 1.46)	0.44	0.42 (−0.09 to 0.92)	−0.09	−0.06 (−0.59 to 0.46)
Resides in city (reference outside city limits, *n* = 92)									
Within city limits (*n* = 205)	0.47**	0.14 (−0.18 to 0.45)	0.43**	0.24 (−0.06 to 0.54)	0.34**	0.30 (−0.02 to 0.61)

*n* = 297 unless otherwise noted.

a Multivariate models adjusted for all covariates.

b Nine missing; coded as median (30 lb).

c Four missing; coded as median ($31,860).

d Four missing; coded as median (high school graduate).

e Six missing; coded as median (2nd trimester).

f Eleven missing; coded with an indicator variable.

* *p* < 0.05.

** *p* < 0.01.

**Table 3:** β coefficients, indicating change in (ln)PCB concentrations, from univariate and multivariate^a^ regression models for selected PCBs.

	CB-118		CB-138/158		CB-153		CB-180	
	β	Adjusted β (95% CI)	β	Adjusted β (95% CI)	β	Adjusted β (95% CI)	β	Adjusted β (95% CI)
Maternal age (per 5 years)	0.17**	0.22** (0.15 to 0.30)	0.31**	0.39** (0.31 to 0.46)	0.36**	0.44** (0.37 to 0.51)	0.48**	0.55** (0.40 to 0.62)
Maternal weight gain^b^ (per 10 lb)	−0.01	−0.01 (−0.05 to 0.03)	0.01	−0.01 (−0.05 to 0.03)	0.01	−0.02 (−0.06 to 0.02)	0.02	−0.02 (−0.06 to 0.02)
Median household income^c^ (per $10,000)	0.04	0.01 (−0.04 to 0.06)	0.06**	0.00 (−0.05 to 0.05)	0.08**	0.01 (−0.03 to 0.05)	0.13**	0.02 (−0.02 to 0.07)
Parity (reference nulliparous, *n* = 123)								
Multiparous (*n* = 174)	0.02	−0.15 (−0.32 to 0.03)	0.18*	−0.13 (−0.30 to 0.03)	0.18*	−0.17* (−0.32 to −0.02)	0.26*	−0.16* (−0.32 to 0.00)
Race (reference Caucasian, *n* = 64)								
Asian (*n* = 23)	0.03	−0.11 (−0.43 to 0.21)	−0.11	−0.33* (−0.64 to −0.02)	−0.04	−0.31* (−0.59 to −0.02)	−0.19	−0.52** (−0.81 to −0.22)
African American (*n* = 210)	−0.11	0.03 (−0.22 to 0.28)	−0.26*	0.10 (−0.14 to 0.34)	−0.33**	0.15 (−0.07 to 0.37)	−0.57**	0.09 (−0.13 to 0.31)
Marital status (reference unmarried, *n* = 198)								
Married (*n* = 99)	0.12	−0.34**(−0.60 to −0.08)	0.28**	−0.34**(−0.58 to −0.09)	0.40**	−0.28* (−0.51 to −0.05)	0.61**	−0.26 (−0.50 to −0.03)
Maternal education^d^ (reference < high school, *n* = 86)								
High school graduate (*n* = 101)	0.03	0.05 (−0.14 to 0.24)	−0.02	−0.02 (−0.21 to 0.16)	0.01	−0.03 (−0.20 to 0.14)	0.00	−0.07 (−0.24 to 0.10)
1–4 years college (*n* = 70)	0.09	0.15 (−0.10 to 0.41)	0.16	0.09 (−0.15 to 0.33)	0.19	0.01 (−0.22 to 0.23)	0.31*	−0.02 (−0.25 to 0.21)
≥ 5 years college (*n* = 40)	0.42**	0.41 (0.04 to 0.78)	0.54**	0.32 (−0.03 to 0.67)	0.70**	0.24 (−0.09 to 0.57)	0.92**	0.22 (−0.11 to 0.56)
Prenatal care^e^ (reference 1st or 2nd trimester, *n* = 281)								
None or 3rd trimester (*n* = 16)	0.04	−0.02 (−0.36 to 0.33)	−0.02	−0.10 (−0.43 to 0.23)	0.04	−0.05 (−0.35 to 0.26)	0.10	0.03 (−0.28 to 0.34)
Smoking during pregnancy (reference nonsmoker, *n* = 242)								
Smoker (*n* = 55)	0.09	0.10 (−0.10 to 0.30)	0.34**	0.30** (0.11 to 0.49)	0.27*	0.21* (0.04 to 0.39)	0.30*	0.24** (0.06 to 0.42)
BMI^f^ (kg/m^2^) (reference normal, *n* = 135)								
Underweight (*n* = 11)	−0.25	−0.24 (−0.58 to 0.10)	−0.23	−0.23 (−0.56 to 0.09)	−0.27	−0.25 (−0.56 to 0.05)	−0.22	−0.19 (−0.49 to 0.12)
Overweight (*n* = 65)	−0.13	−0.07 (−0.43 to 0.29)	−0.29	−0.25 (−0.59 to 0.09)	−0.41	−0.35* (−0.67 to −0.03)	−0.50*	−0.39* (−0.72 to −0.07)
Obese (*n* = 71)	−0.24	−0.20 (−0.57 to 0.16)	−0.49*	−0.48**(−0.83 to −0.14)	−0.64**	−0.62**(−0.94 to −0.30)	−0.74**	−0.67** (−1.00 to −0.34)
Resides in city (reference outside city limits, *n* = 92)								
Within city limits (*n* = 205)	−0.04	0.12 (−0.10 to 0.34)	−0.17	0.06 (−0.15 to 0.27)	−0.22*	0.09 (−0.10 to 0.29)	−0.39**	0.09 (−0.11 to 0.28)

*n*= 297 unless otherwise noted.

a Multivariate models adjusted for all covariates.

b Nine missing; coded as median (30 lbs).

c Four missing; coded as median ($31,860).

d Four missing; coded as median (HS graduate).

e Six missing; coded as median (2nd trimester).

f Eleven missing; coded with an indicator variable.

* *p* < 0.05.

** *p* < 0.01.
